# Infrared FEL-Induced Alteration of Zeta Potential in Electrochemically Grown Quantum Dots: Insights into Ion Modification

**DOI:** 10.3390/nano15201543

**Published:** 2025-10-10

**Authors:** Sukrit Sucharitakul, Siripatsorn Thanasanvorakun, Vasan Yarangsi, Suparoek Yarin, Kritsada Hongsith, Monchai Jitvisate, Hideaki Ohgaki, Surachet Phadungdhitidhada, Heishun Zen, Sakhorn Rimjaem, Supab Choopun

**Affiliations:** 1 Department of Physics and Materials Science, Faculty of Science, Chiang Mai University, Chiang Mai 50200, Thailand; sukrit.sucharitakul@cmu.ac.th (S.S.); kaimuksrps@gmail.com (S.T.); vasan.yarangsi@gmail.com (V.Y.); wivebwc302@hotmail.co.th (S.Y.); kritsada354@gmail.com (K.H.); surelity@gmail.com (S.P.); sakhorn.rimjaem@cmu.ac.th (S.R.); 2 Physics of Low-Dimensional Systems for Opto-Electronic Applications, Department of Physics and Materials Science, Faculty of Science, Chiang Mai University, Chiang Mai 50200, Thailand; 3 Research Unit for Development and Utilization of Electron Linear Accelerator and Ultrafast Infrared/Terahertz Laser, Chiang Mai University, Chiang Mai 50200, Thailand; 4 Thailand Center of Excellence in Physics, Ministry of Higher Education, Science, Research and Innovation, Bangkok 10400, Thailand; 5 School of Physic, Institute of Science, Suranaree University of Technology, 111 University Avenue, Muang, Nakhon Ratchasima 30000, Thailand; monchai@g.sut.ac.th; 6 Institute of Advanced Energy, Kyoto University, Gokasho, Uji 611-0011, Kyoto, Japan; ohgaki.hideaki.2w@kyoto-u.ac.jp (H.O.); zen@iae.kyoto-u.ac.jp (H.Z.); 7 PBP-CMU Electron Linac Laboratory, Plasma and Beam Physics Research Facility, Department of Physics and Materials Science, Faculty of Science, Chiang Mai University, Chiang Mai 50200, Thailand

**Keywords:** quantum dots, free electron laser, irradiation, electrochemical process, optoelectronics

## Abstract

This study explores the use of mid-infrared (MIR) free-electron laser (FEL) irradiation as a tool for tailoring the surface properties of electrochemically synthesized TiO_2_—graphene quantum dots (QDs). The QDs, prepared in colloidal form via a cost-effective electrochemical method in a KCl—citric acid medium, were exposed to MIR wavelengths (5.76, 8.02, and 9.10 µm) at the Kyoto University FEL facility. Post-irradiation measurements revealed a pronounced inversion of zeta potential by 40–50 mV and approximately 10% reduction in hydrodynamic size, indicating double-layer contraction and ionic redistribution at the QD—solvent interface. Photoluminescence spectra showed enhanced emission for GQDs and TiO_2_/GQD composites, while Tauc analysis revealed modest bandgap blue shifts (0.04–0.08 eV), both consistent with trap-state passivation and sharper band edges. TEM confirmed intact crystalline structures, verifying that FEL-induced modifications were confined to surface chemistry rather than bulk lattice damage. Taken together, these results demonstrate that MIR FEL irradiation provides a resonance-driven, non-contact method to reorganize ions, suppress defect states, and improve the optoelectronic quality of QDs. This approach offers a scalable post-synthetic pathway for enhancing electron transport layers in perovskite solar cells and highlights the broader potential of photonic infrastructure for advanced nanomaterial processing and interface engineering in optoelectronic and energy applications.

## 1. Introduction

Perovskite solar cells (PSCs) have garnered considerable attention since their first demonstration by Kojima et al. in 2009 [1], owing to their rapid increase in power conversion efficiency (PCE) from 3.8% to over 25% within a decade [2]. The appeal of PSCs lies in their low-temperature, solution-processable fabrication, enabling cost-effective and scalable manufacturing. However, the long-term stability of PSCs remains a critical barrier to commercialization, largely due to defect formation and inefficient charge extraction at the interfaces between the perovskite absorber and the transport layers [3].

The electron transport layer (ETL), often composed of titanium dioxide (TiO_2_), plays a central role in facilitating photo-generated electron extraction and reducing recombination losses. Despite favorable band alignment and transparency, TiO_2_ suffers from low carrier mobility and UV sensitivity, necessitating modifications to enhance its performance. Incorporation of nanomaterials such as graphene quantum dots (GQDs) has emerged as a promising route to passivate surface defects and improve electronic properties at the ETL/perovskite interface [4,5].

GQDs, with their sp^2^-hybridized carbon structure and size-tunable band gaps, exhibit excellent chemical stability, high conductivity, and strong quantum confinement effects [6,7]. In TiO_2_—graphene hybrid structures, GQDs can improve charge transport and reduce carrier recombination by forming effective heterojunctions, thereby enhancing the PCE, fill factor (*FF*), and short-circuit current (*Jsc*) of PSCs [3,8,9,10].

In our recent work [11], we developed a one-step electrochemical synthesis of TiO_2_-GQDs nanocomposite quantum dots (QDs) in colloidal form and demonstrated their successful integration as part of the mesoscopic ETL using a titanium tetraisopropoxide (TTIP) scaffold. This scalable method yielded significant improvements in device performance metrics—most notably increasing the PCE from 12.0% to 15.1%—as well as reduced trap density and enhanced grain size, attributed in part to the passivation effects of K^+^ and Cl^−^ ions from the electrochemical environment.

Expanding upon this foundation, the current study explores the impact of mid-infrared (MIR) free electron laser (FEL) irradiation on the surface charge behavior of these electrochemically synthesized TiO_2_-GQDs QDs. Photon-induced surface modification is of particular interest because zeta potential plays a critical role in governing colloidal stability, surface dipole orientation, and charge transfer at interfaces, which are key parameters in many optoelectronic and energy-related systems. Using MIR wavelengths (5.76, 8.02, and 9.10 μm based of known Fourier transform infrared spectroscopy (FTIR) bonds in Ti–O–C, C = O, and C–OH previously reported [12,13]) irradiation via FEL, we observed an inversion of zeta potential up to 40–50 mV post-irradiation. This result indicates a pronounced reorganization of surface ions and dipole alignment, highlighting FEL irradiation as a precise, non-contact means of tuning nanoscale interfacial properties.

To the best of our knowledge, this is the first report of FEL-induced zeta potential inversion in QDs intended for optoelectronic applications. This work presents an innovative route for tuning surface charge behavior through photonic stimuli, providing a new degree of freedom for optimizing the charge extraction interface in next-generation PSCs.

## 2. Materials and Methods

### 2.1. Quantum Dots Synthesis

The quantum dots used in this study—including TiO_2_ QDs, graphene quantum dots (GQDs), and heterostructured nanocomposites—were synthesized via a solution-based electrochemical method, adapted from our previous work [11]. The synthesis was performed in a mixed electrolyte containing 0.3 M potassium chloride (KCl) and 0.1 M citric acid dissolved in deionized (DI) water.

**Figure 1:**
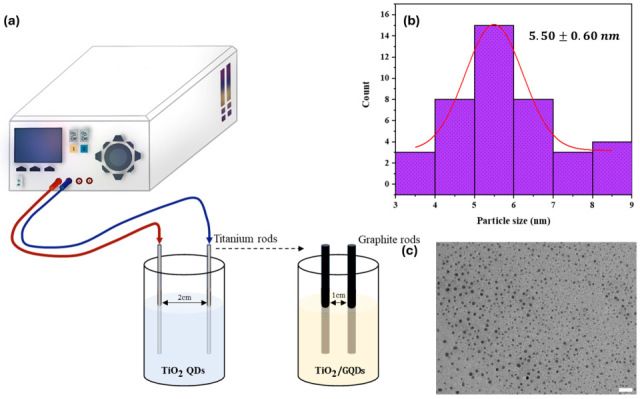
(**a**) Schematic of the electrochemical synthesis setup used for preparing TiO_2_ and TiO_2_/GQDs quantum dots (QDs). A DC power supply drives the reaction between titanium or graphite electrodes in a KCl—citric acid electrolyte, with electrode spacings of 2 cm for TiO_2_ QDs and 1 cm for TiO_2_/GQDs composites. (**b**) Histogram of particle size distribution obtained from ImageJ analysis of 40 individual QDs, fitted with a Gaussian curve, yielding an average diameter of 5.50 ± 0.60 nm with red line as normal distribution fit. (**c**) Representative TEM image of the as-synthesized QDs used for the size analysis (white scale bar for 20 nm reference).

Cleaned titanium and graphite rods served as electrodes in a two-electrode symmetric configuration. A constant direct current (DC) voltage of 10–15 V was applied across the electrodes for 30 to 60 min, depending on the synthesis condition. No reference electrode was used; thus, all voltages are reported with respect to the corresponding symmetric electrode configuration as shown in Figure 1a.

The particle size distribution was determined by analyzing 40 individual quantum dots from representative TEM images (bottom right) using ImageJ ver. 1.51 on part of image in Figure 1c. Each particle’s diameter was manually measured, and the resulting dataset was fitted with a Gaussian distribution. The analysis yielded an average particle diameter of 5.50 nm with a standard deviation of approximately 0.60 nm as shown in Figure 1b, indicating a relatively narrow and uniform size distribution for the electrochemically synthesized TiO_2_/GQDs nanocomposites. This error corresponds to the actual statistical spread of particle sizes, not instrumental error. The following electrode configurations were employed to obtain different QD types:

**TiO_2_ QDs**: Synthesized using two titanium electrodes (E vs. Ti).
**GQDs**: Synthesized using two graphite electrodes (E vs. graphite).
**Ti-GQDs**: Synthesis initiated with titanium electrodes, followed by graphite electrodes.

The resulting colloidal dispersions were directly collected for further surface modification studies. The presence of K^+^ and Cl^−^ ions in the electrolyte served not only as conductive carriers but also played a role in stabilizing the surface of the QDs during growth, as previously reported.

### 2.2. FEL MIR Beam Preparation

MIR-FEL pulses were generated at the Kyoto University Free Electron Laser (KU-FEL) facility [14] using a high-power, tunable MIR-FEL system covering the wavelength range of 3.4 to 26 µm. In this study, the FEL was operated in narrow-band mode (bandwidth ~1% FWHM) to selectively excite vibrational modes relevant to specific surface functional groups on TiO_2_—graphene quantum dot (QD) nanocomposites. The electron beam was produced by a 4.5-cell thermionic S-band RF gun equipped with a LaB_6_ cathode, generating macro-pulses of approximately 7 µs duration with a kinetic energy of 8.3 MeV. After emission, the beam was transported through a dog-leg section comprising two dipole magnets, three quadrupole magnets, and a beam-slicing slit to filter and shape the pulse. Further acceleration was achieved using a traveling-wave accelerator tube, bringing the beam to a maximum energy of 40 MeV. Both the RF gun and accelerator tube were independently powered by 2856 MHz microwave klystrons [14].

Following beam optimization via additional dipole and quadrupole magnets, the electron beam was injected into a 1.8 m hybrid undulator. Spontaneous emission was initiated within the undulator and amplified inside an optical cavity via successive interactions with incoming electron bunches. After 100 to 200 round trips, the stored light intensity in the cavity increased by more than six orders of magnitude, achieving laser oscillation and saturation. For this experiment, the FEL was tuned to generate a narrow bandwidth spectrum with central wavelengths of 5.76 µm, 8.02 µm, and 9.10 µm, corresponding to the vibrational bands of Ti–O–C, C = O, and C–OH groups identified in prior FTIR analysis [12,13].

**Figure 2:**
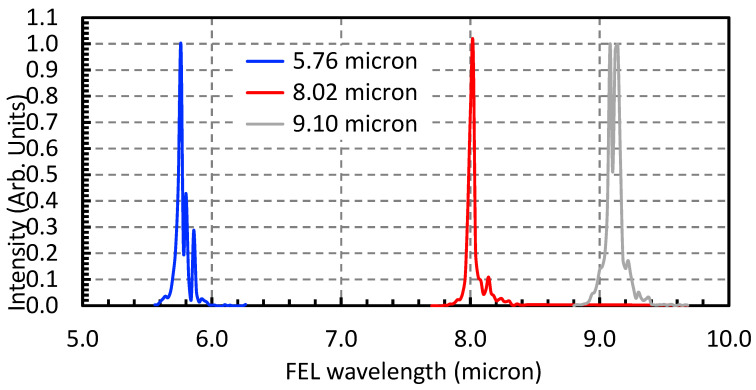
Normalized spectra of MIR FEL used in TiO_2_—graphene quantum dot (QD) irradiation experiments.

To minimize thermal effects while maintaining spectral selectivity, the FEL was operated to deliver macro-pulse energy of 10–40 mJ at each wavelength to the user station. Beam alignment, spectral bandwidth, and pulse energy were carefully optimized and confirmed prior to irradiation, ensuring stable and reproducible MIR exposure suitable for controlled surface modification of colloidal QDs. The FEL beam was focused near the center of the colloid container while shaken in the rotator. The spectra of the MIR FEL used in the TiO_2_—graphene QDs irradiation experiments are shown in Figure 2. Note that each spectrum, corresponding to one of the three central wavelengths, was generated separately in individual irradiation experiments. They are presented together in a single figure to facilitate illustration and comparison.

### 2.3. FEL MIR Irradiation

The synthesized TiO_2_—graphene QDs nanocomposites were subjected to MIR irradiation using the KU-FEL. The objective was to investigate photon-induced modifications in surface chemistry and ion distribution under high-intensity, tunable infrared exposure. Colloidal QD samples were prepared in deionized water containing 0.3 M KCl to ensure colloidal stability during transport and irradiation.

Irradiation was carried out at three selected FEL central wavelengths—5.76 μm, 8.02 μm, and 9.10 μm—chosen to target specific vibrational modes corresponding to Ti–O–C, C = O, and C–OH bonding within the QD structures based on previously reported FTIR results [12,13]. Each sample was exposed for 10 min under high-power pulse operation, maintaining beam parameters consistent with safe colloidal handling.

Post-irradiation, the zeta potential of the colloidal QDs was re-evaluated, revealing a substantial inversion of surface charge by approximately 40–50 mV. This indicated a marked reconfiguration in the ionic environment at the nanocomposite surface. The results suggest that FEL-based MIR exposure offers a controllable route to modify QD surface states, potentially improving charge extraction characteristics when applied in photovoltaic or optoelectronic devices. These findings open new avenues for light-driven post-synthetic processing of nanomaterials tailored for energy applications.

## 3. Results

### 3.1. Quantum Dot Growth

The electrochemical synthesis method employed in this study successfully yielded four distinct types of quantum dots (QDs): TiO_2_ QDs, graphene quantum dots (GQDs), and heterostructured composites Ti–GQDs. The process, performed in a two-electrode symmetric configuration without a reference electrode, utilized a potassium chloride (KCl) and citric acid aqueous electrolyte, offering a cost-effective, scalable, and environmentally benign route to generate colloidal QDs. The synthesis conditions—including applied DC voltage (10–15 V), reaction time (30–60 min), and electrode switching protocol—were systematically varied to explore the impact on QD composition and stability.

Figure 1 visually presents the outcome of these syntheses, highlighting observable differences in colloidal color, transparency, and dispersion characteristics. TiO_2_ QDs appeared milky white to pale yellow, consistent with the formation of oxide nanoparticles in the sub-10 nm range, while GQDs exhibited a yellow-brown tint typical of carbon-based nanodots. The heterostructured QDs—Ti–GQDs showed intermediate shades with enhanced translucency, indicating successful hybridization of oxide and graphene components. These visual cues, although qualitative, are indicative of variations in particle size, surface oxidation, and interfacial chemistry.

The electrolyte composition, particularly the presence of 0.3 M KCl, played a dual role in the synthesis process. First, it served as a conductive medium to facilitate ion transport and electron transfer reactions at the electrode surfaces. Second, the K^+^ and Cl^−^ ions acted as surface passivating agents, stabilizing the colloidal QDs during nucleation and growth. This stabilization is essential for preventing aggregation and promoting uniform particle size distribution, especially for the graphene—TiO_2_ heterostructures. Citric acid in the electrolyte further contributed to colloidal stability by coordinating surface metal centers and imparting negative surface charges via carboxylate groups.

Among the heterostructured variants, the Ti–GQDs configuration—where the titanium electrode was used first followed by graphite—yielded QDs with improved dispersibility and colloidal lifetime compared to the reverse order. This observation suggests that the initial formation of TiO_2_ nuclei provides a favorable template for subsequent carbon integration, possibly forming Ti–O–C bridges that enhance charge delocalization and electronic coupling between the oxide and graphene phases.

Preliminary characterization, including zeta potential measurements prior to FEL irradiation, confirmed that all synthesized QD variants exhibited stable colloidal behavior with negative surface charges in the range of −10 to −20 mV. These baseline values are crucial for assessing the extent of photo-induced surface modification in later stages of the study. Additionally, the simplicity and reproducibility of the electrochemical method make it suitable for high-throughput synthesis and integration with downstream photonic treatments, such as the FEL-based surface tuning described in the following sections.

In summary, the electrochemical growth process provided a robust platform for producing colloidal QDs with tunable composition, surface functionality, and colloidal stability. The method’s versatility in generating both pure and heterostructured QDs lays the foundation for exploring advanced optoelectronic applications, especially when combined with post-synthetic surface engineering via MIR FEL irradiation.

### 3.2. FEL-Induced Surface Modification

Following synthesis, the colloidal QDs—with emphasis on the Ti–GQDs heterostructure—were subjected to MIR irradiation using the Free Electron Laser (FEL) at Kyoto University. The irradiation process was designed to probe photon-induced changes in surface chemistry, electrostatic environment, and bonding configurations of the QDs. This approach leverages the tunability and high peak power of FEL radiation to selectively excite specific vibrational modes associated with surface functional groups, potentially triggering surface passivation or bond reconfiguration without altering the core crystal structure.

The colloidal QDs were irradiated at three discrete FEL wavelengths: 5.76 µm, 8.02 µm, and 9.10 µm. These correspond to known vibrational absorptions in the QD system, specifically the Ti–O–C asymmetric stretch, C = O stretching in carboxylic groups, and C–OH deformation modes, respectively [12,13]. The wavelength-targeted approach ensures that the FEL energy is deposited preferentially into surface functionalities rather than inducing bulk heating or ablation. Each irradiation session lasted 10 min under macro-pulse operation, with pulse energies maintained between 30 and 40 mJ, except for the FEL with a central wavelength of 9.10 µm, whose macro-pulse energy was approximately 15 µm. to ensure sufficient energy delivery while avoiding colloidal destabilization.

**Zeta potential measurements**, conducted before and after FEL exposure, provided a direct metric for assessing changes in surface charge. Remarkably, all irradiated QD samples exhibited a significant inversion in zeta potential, shifting from initial values of approximately −10 mV to +30–40 mV. This dramatic reversal indicates a fundamental change in the surface ion distribution, likely caused by the reorientation or removal of surface-bound ionic species, or by photon-induced generation of new surface dipoles. The magnitude and direction of the shift were consistent across all tested wavelengths.

Interestingly, no visual aggregation or precipitation was observed post-irradiation, and the colloidal suspensions retained their stability for over 3 weeks of observation, implying that the FEL-induced changes did not compromise colloidal integrity. Instead, the irradiation appears to have refined the electrostatic profile of the QDs by modifying their surface dipole orientation and neutralizing labile anionic groups. The shift from negative to positive zeta potential also suggests a transition from a predominantly anionic outer shell (e.g., citrate-bound carboxylates) to a cation-enriched surface, possibly involving K^+^ ion trapping or field-driven reorganization under pulsed laser excitation.

These findings provide compelling evidence that FEL-based MIR exposure offers a non-contact, wavelength-selective strategy for tuning QD surface properties, particularly for optoelectronic interfaces where charge extraction and band alignment are sensitive to surface dipoles. The observed inversion of surface potential suggests potential benefits for reducing interfacial recombination and improving dipole alignment in electronic and optoelectronic systems. Additionally, FEL irradiation could be integrated as a post-synthesis treatment in roll-to-roll or inkjet-printed QD processing, particularly in scalable manufacturing environments.

**Figure 3:**
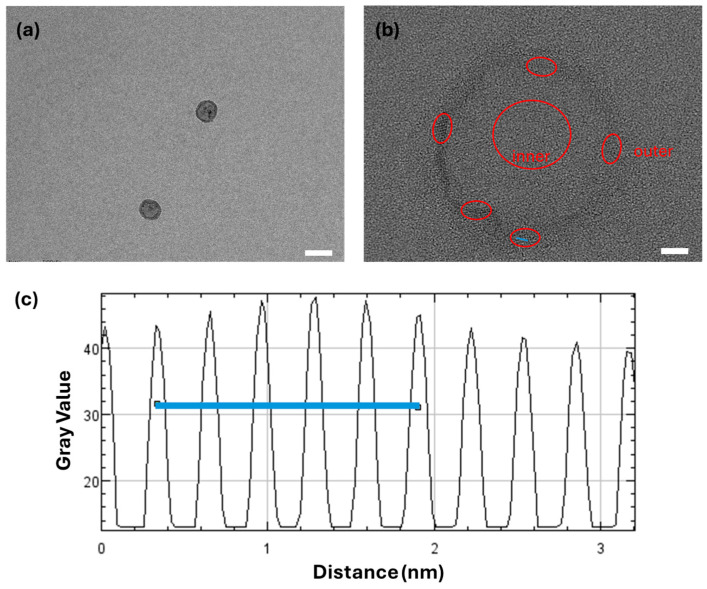
(**a**) HD-TEM image of the treated TiO_2_ QDs (white scale bar corresponds 50 nm), (**b**) zoomed in and modified version of the QD (white scale bar corresponds to 5 nm) with heterostructure highlighted in red, and (**c**) profile mapping of the blue line in (**b**).

High-resolution transmission electron microscopy (HRTEM) images of the irradiated QDs confirmed that the core crystal structure remained intact after FEL exposure as shown in Figure 3. Lattice fringes corresponding to the (002) planes of graphene (~0.326 nm) and the (110) and (004) planes of TiO_2_ (rutile and anatase, ~0.32 nm and 0.245 nm, respectively) were still clearly observed post-irradiation, indicating that the FEL pulses did not degrade or amorphize the QD crystalline domains. These findings suggest that MIR irradiation induces surface-level functional modifications rather than structural damage, thus preserving the optoelectronic integrity of the QDs.

## 4. Data Analysis

The inversion of zeta potential observed post-irradiation marks a significant alteration in the electrostatic surface profile of the TiO_2_—graphene QDs nanocomposites. Prior to irradiation, the colloidal system exhibited a negatively charged surface, indicative of surface-adsorbed anionic species or polar oxygen-containing functional groups. The zeta potential (*ζ*), a key parameter for colloidal stability and surface charge, can be related to the measured electrophoretic mobility (*μ_e_*) through the Smoluchowski relation:

$$\zeta =\frac{4\pi \eta \mu_{e}}{\epsilon }$$

where *η* is the solvent viscosity and ε its dielectric constant. In parallel, the hydrodynamic size ($D_{H}$) of the particles, obtained from dynamic light scattering (DLS), provides complementary insight into changes in solvation shells and surface packing, and is expressed as:

$$D_{H}=\frac{k_{B}T}{3\pi \eta D_{t}}$$

with $k_{B}$ being Boltzmann’s constant, *T* the absolute temperature, and $D_{t}$ the translational diffusion coefficient.

**Figure 4:**
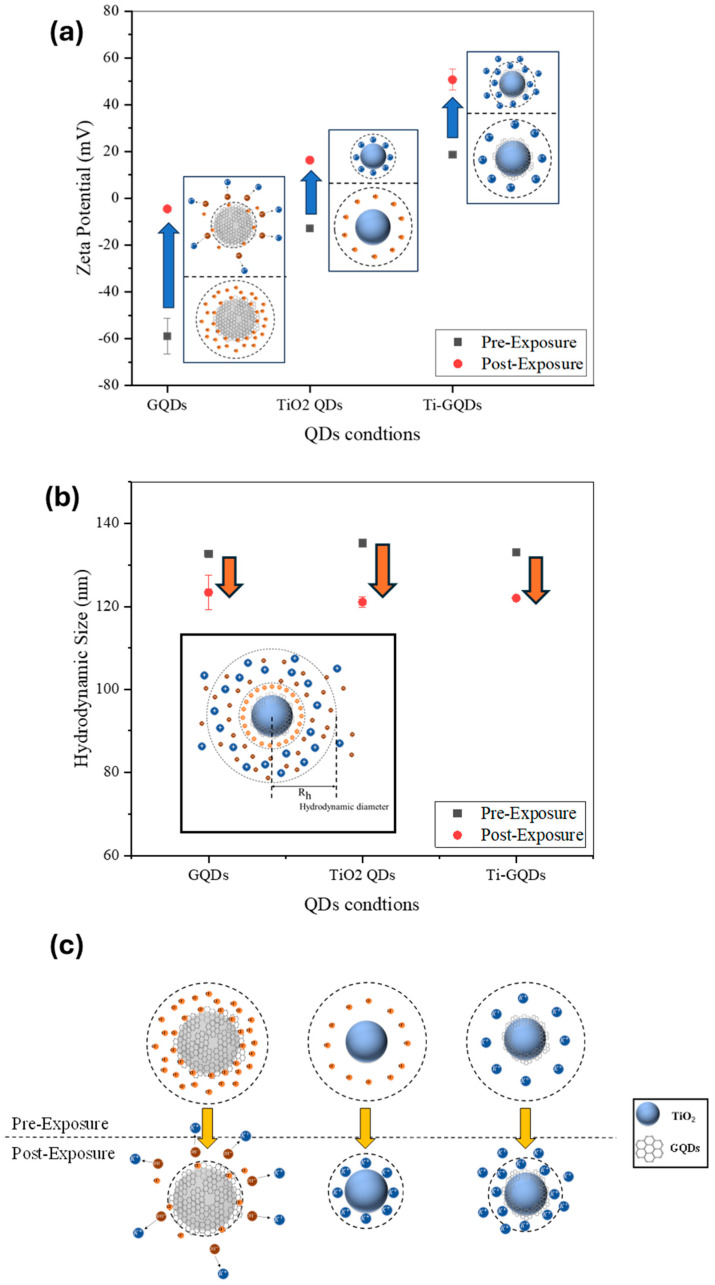
(**a**) Zetapotential, (**b**) Hydrodynamics Size of QDs pre and post exposure and (**c**) graphical schemes of changes happening during exposure.

Following exposure to narrow-band MIR FEL pulses, this surface charge reversed direction, shifting by approximately 40–50 mV toward a net positive potential. Such a reversal is not a trivial modulation but instead implies a fundamental change in the dominant surface dipole orientation or ionic configuration at the QD—solvent interface as shown in Figure 4.

Figure 4 summarizes the FEL-induced changes in the colloidal QDs. Figure 4a highlights the inversion of zeta potential from negative to positive values, consistent with ion redistribution and dipole reorientation at the surface. Figure 4b presents the hydrodynamic size before and after irradiation, showing a slight but systematic reduction, indicative of a more compact solvation shell. Figure 4c schematically illustrates the proposed mechanism, where FEL excitation disrupts adsorbed anionic groups, promotes K^+^ coordination, and reorganizes surface dipoles, ultimately leading to a stabilized and positively charged colloidal interface. This phenomenon can be interpreted in terms of resonant vibrational excitation of surface functional groups, which likely initiates transient polarization and reorganization of adsorbed ions. The ion rearrangement may be driven by local heating at submicron scale, cooperative oscillation of bonded moieties (e.g., Ti–O–C and C–OH), or dipole flipping under strong field gradients associated with FEL pulses. Moreover, the presence of coordinating ions from the electrochemical growth medium—specifically K^+^ and Cl^−^—may amplify these effects by contributing to dynamic ion-exchange equilibria on the QD surface. These modifications support a shift toward more compact and possibly protonated surface environments, consistent with the observed reversal in surface potential. It is also noteworthy that the irradiated dispersions exhibited excellent colloidal stability for an extended period. No visible precipitation or phase separation was observed after more than three weeks of storage at ambient conditions. The ζ-potential values remained consistently positive (≈+30 mV) throughout this period, indicating stable electrostatic repulsion between particles. Optical inspection revealed no discernible change in dispersion transparency or color, further confirming the persistence of the FEL-induced surface modification as highlighted in Table 1. This stability is comparable to, or exceeds, that typically reported for chemically passivated TiO_2_–GQD colloids, highlighting the robustness of the photonic treatment.

To provide a quantitative framework, the observed zeta potential changes can also be described within the Debye–Hückel approximation, which relates the potential (ζ) at the slipping plane (*d*) to the surface potential (*ψ*_0_) and the ionic strength (*I*) of the medium:

$$\zeta \approx \psi_{0}e^{-\kappa d},\;\kappa^{-1}=\sqrt{\frac{\epsilon k_{B}T}{2N_{A}e^{2}I}}$$

where *d* is the distance from the particle surface to the slipping plane, *κ*^−1^ is the Debye length, *I* the ionic strength, *ε* the dielectric constant of the medium, $k_{B}$ Boltzmann’s constant, T the absolute temperature, $N_{A}$ Avogadro’s number, and e the elementary charge. This description highlights how FEL-induced ion redistribution and dipole reorientation effectively alter *ζ* by modifying the local ionic strength and double-layer thickness around the QDs. Taken together, the concurrent inversion of *ζ* and reduction in $D_{H}$ can be rationalized within a unified framework of double-layer modification. In the Debye–Hückel picture, FEL irradiation perturbs the ionic atmosphere near the QD surface, effectively reducing the Debye length (*κ*^−1^) and shifting the slipping plane. This contraction of the electrostatic double layer leads to a higher apparent *ζ* potential while simultaneously tightening the solvation shell, consistent with the smaller hydrodynamic diameters observed by DLS. This is most likely due to increased ionic strength in the solution (*I*). Thus, both electrokinetic and size measurements converge to indicate that FEL exposure reorganizes the interfacial environment into a more compact, cation-stabilized configuration.

**Figure 5:**
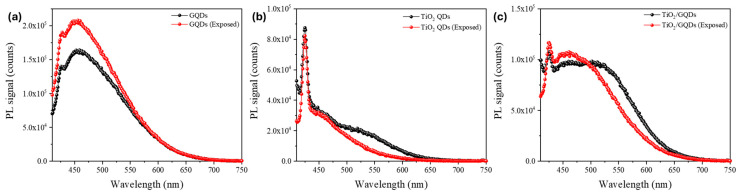
Steady-state photoluminescence (PL) spectra of (**a**) GQDs, (**b**) TiO_2_ QDs, and (**c**) TiO_2_/GQD nanocomposites before FEL irradiation (black) and after FEL irradiation (red).

Figure 5 presents the PL spectra of GQDs, TiO_2_ QDs, and TiO_2_/GQD nanocomposites before (black) and after FEL irradiation (red). GQDs exhibit enhanced PL intensity with negligible spectral shift, indicating trap-state passivation. TiO_2_ QDs show PL quenching, consistent with enhanced non-radiative pathways at oxide surfaces. The TiO_2_/GQD heterostructures display modest broadband PL enhancement, reflecting improved interfacial charge transfer. Together, these results support the conclusion that FEL irradiation reorganizes surface states in a composition-dependent manner without altering the emissive core structures. For GQDs, the PL intensity increased by approximately 25–35% with no resolvable spectral shift (|Δλ| ≲ 5 nm), indicating suppression of non-radiative trap states while preserving the emissive core. In contrast, TiO_2_ QDs exhibited a 10–20% reduction in PL without a peak shift, consistent with enhanced non-radiative recombination at oxide surface states. The TiO_2_/GQD heterostructures displayed an intermediate response, with modest broadband PL enhancement and unchanged peak position, suggesting improved charge transfer across the oxide–graphene interface. These observations support the conclusion that FEL exposure alters the balance of radiative and non-radiative pathways in a composition-dependent manner, primarily through surface-state reorganization rather than bulk structural changes.

**Figure 6:**
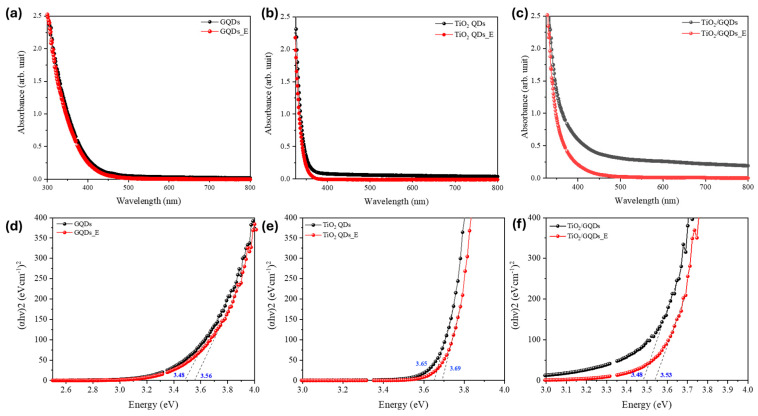
UV—Vis absorption spectra (**a**–**c**) and corresponding Tauc plots (**d**–**f**) of (**a**,**d**) TiO_2_/GQDs, (**b**,**e**) TiO_2_ QDs, and (**c**,**f**) GQDs before FEL irradiation (black) and after FEL irradiation (red). A slight bandgap blue shift is observed in all cases (e.g., TiO_2_/GQDs: 3.48 → 3.53 eV; TiO_2_ QDs: 3.65 → 3.69 eV; GQDs: 3.48 → 3.56 eV), consistent with trap-state passivation and sharpening of the absorption edge without altering crystalline structure.

The optical bandgap of the quantum dots was estimated using the Tauc relation for a direct allowed transition, expressed as:

$${\left(\alpha h\nu \right)}^{1/n}=A\left(h\nu -E_{g}\right),$$

where α is the absorption coefficient, hν is the photon energy, A is a proportionality constant, and Eg is the optical bandgap energy. Plots of ${\left(\alpha h\nu \right)}^{2}$ versus hν were constructed (assuming *n* = 1/2 for allowed direct transition), and the linear region near the absorption edge was extrapolated to the energy axis to obtain the bandgap. This direct transition model is appropriate for both TiO_2_ quantum dots and graphene quantum dots and was used consistently across all samples before and after FEL exposure. The corresponding Tauc plots in Figure 6 further corroborate this interpretation. The optical bandgap of TiO_2_/GQDs increased from ~3.48 to 3.53 eV, TiO_2_ QDs from ~3.65 to 3.69 eV, and GQDs from ~3.48 to 3.56 eV. These small but systematic blue shifts (0.04–0.08 eV) indicate a reduction in sub-bandgap tail states and sharpening of the absorption edge, consistent with trap-state passivation and the PL enhancements observed in Figure 5. Importantly, the absence of significant band-edge displacement confirms that the crystalline cores remain intact after FEL exposure, in agreement with TEM lattice-fringe observations. Compared to conventional post-synthetic passivation techniques such as chemical ligand exchange, thermal annealing, or UV—ozone treatment, FEL irradiation offers several unique advantages. While chemical treatments can introduce unwanted residues or require multi-step procedures, and thermal treatments risk altering the core crystal structure, FEL exposure provides a resonant, non-contact mechanism that selectively modifies surface states through vibrational excitation. The observed PL enhancement and bandgap blue shifts are on par with values typically achieved via ligand exchange or UV—ozone treatments, but without chemical additives or elevated temperatures. This highlights FEL irradiation as a clean and scalable alternative for surface passivation in QD systems.

DLS revealed approximately 10% reduction in hydrodynamic size after FEL exposure, consistent with a more compact solvation shell. TEM confirmed that lattice fringes of both anatase TiO_2_ and graphitic domains remained intact, showing that the treatment affects only surface chemistry and electrostatics without damaging the crystalline cores. Optical measurements reinforced this picture: PL enhancement in GQDs and TiO_2_/GQDs, together with modest bandgap blue shifts in the Tauc plots, point to trap-state suppression and sharper band edges. These trends align with the ζ-potential inversion in Figure 4, all attributable to FEL-induced increases in ionic strength. A higher ionic strength compresses the electrostatic double layer by shortening the Debye length, which shifts the slipping plane closer to the QD surface and reduces the contribution of loosely bound solvent molecules to the hydrodynamic radius. At the same time, stronger screening and cation coordination stabilize surface functional groups, neutralize anionic defects, and reduce sub-bandgap tail states. Taken together, Figure 4, Figure 5 and Figure 6 demonstrate that MIR FEL irradiation provides a resonance-driven, non-contact route for post-synthetic surface engineering, yielding more stable colloids, reduced recombination, and improved interfacial quality for perovskite solar cells, while offering a scalable strategy for tuning solution-processed quantum nanostructures.

## 5. Conclusions

This work demonstrates a novel photonic route to tailoring the surface properties of electrochemically synthesized TiO_2_—graphene quantum dots through MIR-FEL irradiation. Resonant excitation of surface vibrational modes produced a striking inversion of the zeta potential by 40–50 mV and a slight reduction in hydrodynamic size, signifying double-layer contraction and ionic redistribution at the QD—solvent interface. TEM confirmed that crystalline lattices remained intact, indicating that FEL treatment modifies only surface chemistry while preserving structural integrity. Complementary PL and Tauc analyses revealed enhanced emission intensity, small bandgap blue shifts, and reduced Urbach tails, all consistent with trap-state passivation and sharper band edges. The inversion of ζ-potential from negative to positive values following FEL exposure has direct implications for electron transport layer design in optoelectronic devices. A positively charged surface can reduce interfacial trap-assisted recombination by passivating negatively charged defect sites and promoting favorable dipole alignment at the ETL/perovskite interface. In previous studies on TiO_2_–GQD-modified ETLs, such surface engineering has led to efficiency improvements of 2–4% absolute, attributed to enhanced electron injection and reduced interfacial losses [3,8,9,10]. Although no device measurements are presented here, the magnitude of the observed ζ-potential shift (≈40–50 mV) is comparable to those reported in systems that exhibited similar improvements. This suggests that MIR-FEL treatment could serve as a non-chemical route to tune interfacial energetics in ETLs for perovskite and related devices.

Taken together, these findings establish that FEL-induced ionic reorganization compacts the electrostatic double layer, suppresses defect states, and improves the optoelectronic quality of the nanocomposites without resorting to additional chemical treatments. This resonance-driven, non-contact method can be readily integrated into solution-phase processing pipelines, offering a scalable post-synthetic strategy to enhance electron transport layers in perovskite solar cells. More broadly, the results highlight the potential of photonic infrastructure such as KU-FEL to drive innovation in colloidal nanomaterials and enable precise, light-based control of interfacial energetics for next-generation optoelectronic devices. Beyond photovoltaics, this approach may also benefit related fields such as photocatalysis, sensing, and electrochemical energy storage, where surface states and interfacial charge transfer critically determine performance.

## Figures and Tables

**Table 1:** Data extracted from Figure 3.

Condition	Pre-Exposure		Post-Exposure	
	Hydrodynamic Size (nm)	Zeta Potential (mV)	Hydrodynamic Size (nm)	Zeta Potential (mV)
GQDs	132.72±0.87	−58.92±7.68	123.37±4.22	−4.57±0.07
TiO_2_ QDs	135.27±0.38	−12.84±1.47	121.05±1.32	16.29±0.88
Ti-GQDs	133.01±0.34	18.70±0.29	122.01±0.30	50.72±4.46

## Data Availability

The original contributions presented in this study are included in the article. Further inquiries can be directed to the corresponding author.
